# Monomethyl Fumarate Modulates Iron Metabolism and Mitochondrial Function in Microglia with Implications for Multiple Sclerosis Progression

**DOI:** 10.1007/s10571-026-01775-x

**Published:** 2026-07-20

**Authors:** Justus Dann, Katharina Klöster, Ulas Ceylan, Neele Heitmann, Britta Eggers, Svitlana Rozanova, Martin Eisenacher, Katrin Marcus-Alic, Konstanze F. Winklhofer, Ralf Gold, Simon Faissner

**Affiliations:** 1 Department of Neurology, Ruhr-University Bochum, St. Josef-Hospital, Gudrunstr. 56, 44791 Bochum, Germany; 2 Medizinisches Proteom-Center, Medical Faculty, Ruhr-University Bochum, Bochum, Germany; 3 Medical Proteome Analysis, Center for Protein Diagnostics (PRODI), Ruhr-University Bochum, Bochum, Germany; 4 Core Unit for Bioinformatics (CUBiMed.RUB), Medical Faculty, Ruhr-University Bochum, Bochum, Germany; 5 Department Molecular Cell Biology, Institute of Biochemistry and Pathobiochemistry, Ruhr- University Bochum, Bochum, Germany

**Keywords:** Progressive multiple sclerosis, Microglia, Neuroinflammation, Mitochondria, Neuroprotection

## Abstract

Fumaric acid esters have proven to be effective medications in relapsing-remitting multiple sclerosis with neuroprotective effects. In this study, we investigated the impact of fumaric acid esters on primary murine microglia in vitro compared to DMSO vehicle control. Monomethyl fumarate (MMF) increased MTT reduction in a dose-dependent manner, whereas dimethyl fumarate (DMF) exhibited a biphasic response with low concentrations enhancing MTT reduction and higher concentrations inducing toxicity. Notably, complementary analyses of cell number and cell death did not reveal differences between MMF-treated and control conditions, indicating that the increased MTT reduction reflects enhanced cellular metabolic activity rather than increased viability. Consistent with this interpretation, MMF-treated cells exhibited higher basal and maximal oxygen consumption, spare respiratory capacity, and ATP production in the Seahorse XF Cell Mito Stress Test. Proteomic analysis did not indicate an upregulation of mitochondrial respiratory chain proteins, but instead suggested a qualitative shift in mitochondrial homeostasis, including increased expression of mitophagy-associated proteins. MMF-treated Nrf2-deficient microglia showed a blunted increase in MTT reduction, suggesting an involvement of Nrf2 in mediating MMF-induced metabolic effects. Additionally, MMF modulated the microglial iron metabolism and reduced the uptake of non-transferrin-bound iron and altered the gene expression of iron transport proteins, promoting a shift toward the uptake of less toxic, transferrin-bound iron. MMF mitigated iron-induced toxicity and was associated with upregulation of the ferroptosis suppressor protein, indicating a protective response to iron overload. Together, these findings suggest that MMF enhances microglial metabolic activity and mitochondrial function while reducing iron-mediated toxicity, thereby contributing to its neuroprotective effects.

**Graphical Abstract:**

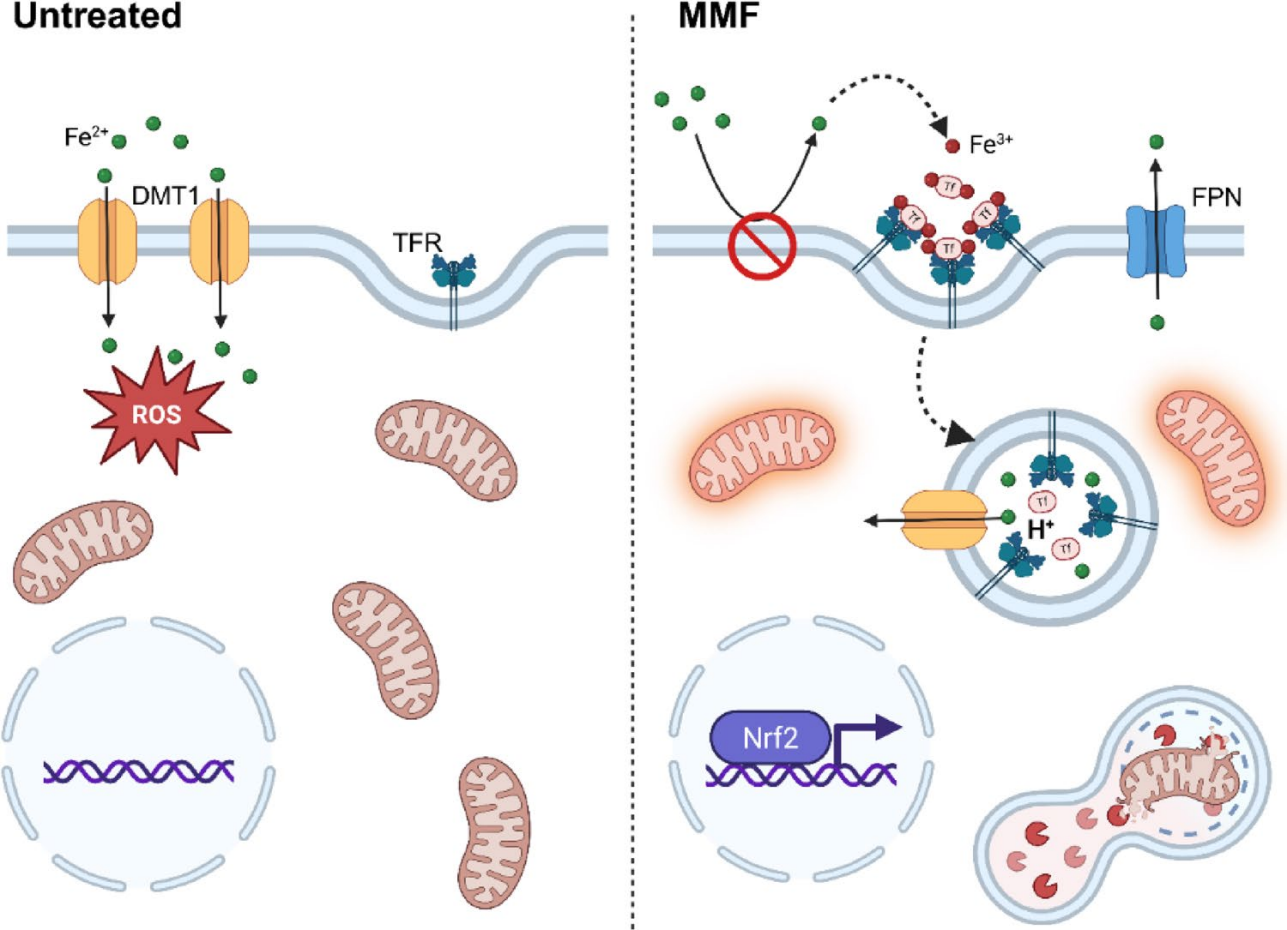

Summary of the proposed cellular effects of monomethyl fumarate in primary murine microglia. Created in BioRender. Faissner (2026). https://help.biorender.com/hc/en-gb/articles/17605511350685

**Supplementary Information:**

The online version contains supplementary material available at 10.1007/s10571-026-01775-x.

## Introduction

Multiple sclerosis (MS) is an autoimmune inflammatory disease of the central nervous system (CNS) that leads to demyelination and neurodegeneration with consecutive disability in the affected individuals (Lassmann et al. 2007). In recent times, great progress has been made regarding the immunomodulatory therapy of the relapsing-remitting form of MS. However, there are still only limited therapeutic options for the progressive phase of the disease since the pathomechanisms of progression are incompletely targeted using established medications (Faissner et al. 2019). According to the current understanding, progressive MS is characterized by a chronic inflammation behind a closed blood-brain barrier involving T-cells, B-cells, activated glial cells, and meningeal inflammation (van Olst et al. 2021). The activated cells, especially reactive microglia, produce reactive oxygen species (ROS), which leads to mitochondrial damage and neurodegeneration (Witte et al. 2014; Yong 2022). Recent MRI studies have shown iron rim lesions to be associated with neurodegeneration in MS. In neuropathologic examinations, iron loaded microglia, the resident immune cells of the CNS, have been identified to be substantially involved in forming these rims (Dal-Bianco et al. 2021).

In the CNS iron is primarily stored in oligodendrocytes (Connor and Menzies 1995) and released upon their destruction during the course of disease. Free iron in its divalent form can catalyze the non-enzymatic formation of hydroxyl radicals in the Fenton reaction (Winterbourn 1995). Given this fact, iron is sequestered inside the cells within ferritin to protect cells from oxidative stress (Connor and Menzies 1995). Divalent iron is directly toxic to neurons, so that recent efforts have focused on reducing iron-mediated neurotoxicity to find new therapeutics for progressive MS (Faissner et al. 2017). Moreover, iron-loaded microglia have also been shown to be neurotoxic in co-culture experiment through ROS release (Yauger et al. 2019).

The fumaric acid esters are well-established drugs for the treatment of relapsing-remitting MS since the approval of dimethyl fumarate (DMF) by the FDA in 2013 following the pivotal phase 3 trial which showed a significant reduction of relapses compared to placebo (Gold et al. 2012). More recently diroximel-fumarate and tegomil fumarate have entered the market. These compounds act solely as prodrugs and do not reach systemic circulation in their parent form, with monomethyl fumarate (MMF) serving as their shared active metabolite (Mrowietz et al. 2018; Rousseau et al. 2023). Besides an immunomodulatory effect on peripheral blood lymphocytes (Mills et al. 2018), also antioxidative and neuroprotective effects of fumaric acid esters have been demonstrated (Linker et al. 2011). The hydroxycarboxylic acid receptor 2 (HCAR2) has been shown to mediate most of the fumarate effects (Chen et al. 2014). Moreover MMF activates the nuclear (erythroid-derived 2)-like 2 (Nrf2) pathway (Linker et al. 2011) which activates several anti-oxidative target genes and also genes of iron metabolism (Kerins and Ooi 2018).

DMF, which has despite being just a prodrug, also been extensively studied in cell culture experiments, can unlike MMF diffuse freely across the cell membrane into the cell. Therefore DMF interacts broadly with various intracellular proteins and thus leads not only to Nrf2 activation, but also to a glutathione depletion, increasing oxidative stress at least temporarily (Albrecht et al. 2012).

The objective of this study is to further elucidate the influence of MMF on microglia with special focus on iron metabolism and mitochondrial function to further understand the underlying mechanisms contributing to its neuroprotective actions.

## Materials and Methods

### Preparation of Glial Cell Cultures

Primary mixed glial cell cultures were prepared from neonatal mice P0-P2. Wildtype mice (C57BL/6) and Nrf2 knockout (Nrf2^−/−^) were obtained from the animal facility of the medical faculty of the Ruhr-University Bochum. Mixed-sex pups were euthanized by decapitation in accordance with approved animal welfare regulations of North Rhine-Westphalia, Germany (no.84-02.04.2017.A132) prior to brain dissection. After removing the meninges from the brain, the cortices were pooled and mechanically dissociated using a pipet. The suspension was passed through a 70 µM cell strainer to remove larger impurities and seeded into poly-D-lysine (Sigma Aldrich, Darmstadt, Germany, Cat.-No. P6407) coated flasks. Cultures were kept in DMEM F12 (Thermo Fisher, Waltham, USA; Cat.-No. 31331093) supplemented with 10% fetal bovine serum (Pan Biotech, Aidenbach, Germany; Cat.-No. P30-3306) and 1% penicillin/streptomycin (Sigma Aldrich; Cat.-No. P0781) at 37 °C and 5% CO_2_.

On the first day after preparation the cellular debris was dislodged by gently clapping against the flasks. From then on, the medium was replaced every 3–4 days.

When the microglia appeared on the confluent layer of astrocytes, which was usually around day 10–14, the flasks were shaken at 220 rpm on an orbital shaker for 2 h and the free-floating microglia were collected, centrifuged and transferred to uncoated cell culture plates. After one hour the medium was replaced, to remove any non-adherent cells yielding a purity of about 95% microglia, as confirmed by Iba1-staining (Fujifilm Wako, Japan; Cat.-No. 019–19741; RRID: AB_839504; see Supplementary Figure S1) and flow cytometric CD11b staining (BD Biosciences, Franklin Lakes, New Jersey, USA; Cat.-No. BDB561688, see Supplementary Figure S2).

The microglia were cultured in a 1:1 mixture of conditioned medium from the mixed glial cell culture flasks and fresh DMEM F12. For iron toxicity studies, the microglia were cultivated in pure DMEM F12 without FBS supplementation to ensure a more consistent toxic effect.

### Cell Treatment and Experimental Timeline

Primary murine microglia were seeded onto cell culture plates 24 h prior to treatments with drugs or iron. Measurements were conducted 24 h after treatment. In selected experiments, cells were pre-treated with the respective drug for 24 h, followed by an additional 24-hour exposure to iron while maintaining the drug treatment, to assess potential protective effects.

MMF (Sigma Aldrich; Cat.-No. 651419-1G) and DMF (Sigma Aldrich; Cat.-No. 242926-25G) were dissolved in DMSO (Sigma Aldrich; Cat.-No. D8418-50ML) as a vehicle due to the limited aqueous solubility of DMF. Stock solutions were stored at −20 °C and freshly thawed and diluted immediately prior to each experiment. Final drug concentrations in the wells after treatment addition were 10, 25, and 50 µM for DMF, and 10, 50, and 100 µM for MMF. The final DMSO concentration in the culture medium was kept at 0.025% across all conditions. Control groups received equivalent amounts of DMSO to account for potential vehicle effects.

A 10 mM iron stock solution was freshly prepared for each experiment by dissolving iron(II) sulfate heptahydrate (Carl Roth, Karlsruhe, Germany; Cat. No. P015.1) in distilled water. The solution was sterile-filtered and subsequently diluted medium prior to application. Final Fe²⁺ concentrations in the wells after treatment addition ranged from 0 to 100 µM.

### MTT Reduction Assay

For the MTT assay primary murine microglia were cultured at density of 30,000 per well in transparent flat bottom 96-well plates (Sarstedt, Nuembrecht, Germany; Cat.-No. 83.3924). An MTT stock solution was prepared by dissolving 5 mg/ml MTT (Carl Roth; Cat.-No. 4022.1) in PBS (GIBCO, Thermo Fisher Scientific; Cat.-No. 15326239) and stored at 5 °C protected from light. The MTT working solution was prepared by diluting the MTT stock solution to a final concentration of 0.5 mg/mL in prewarmed medium. The cells were incubated with 100 µL of the working solution for 4 h in a CO_2_ incubator. Then the medium was removed, and the formazan crystals were dissolved in 200 µL DMSO (VWR, Darmstadt, Germany; Cat.-No. 282164 K). Absorbance was measured at 570 nm on a CLARIOstar plate reader (BMG Labtech, Ortenberg, Germany).

### Live Cell Imaging

For live cell imaging studies primary murine microglia were seeded analogous to the MTT assay at a density of 30.000 cells per well on transparent flat bottom 96-well plates. The cells were preincubated with the respective treatments before live cell permeant Hoechst 33,342 (Invitrogen; Cat.-No. H1399) and live cell impermeant propidium iodide (PI; Invitrogen; Cat.-No. P3566) were added to the medium at a final concentration of 1 µM each.

The live cell imaging setup consisted of an Axio Observer Microscope, Axio Cam 512, Definite Focus 2 (all Zeiss, Oberkochen, Germany) and a XL multi S1 dark LS incubator (PeCon, Erbach, Germany). 3 × 3 images were acquired in the center of each well using a 10× objective and subsequently stitched into a single composite image with 1,500-2,000 cells.

ImageJ was used for the automated analysis of the images.

Automated cell counting was performed in ImageJ using the *Analyze Particles* function with predefined threshold values for the PI and Hoechst channels. Nuclei exhibiting colocalization of PI and Hoechst 33,342 signals were classified as dead cells.

### Annexin V Staining

Primary murine microglia were seeded at a density of 150,000 cells per well on a 24-well plate (Sarstedt) and treated for 24 h with MMF 100 µM or control. Afterwards the cells were first washed with warm PBS (GIBCO, Thermo Fisher Scientific; Cat.-No. 15326239) and then detached using Accutase (GIBCO, Thermo Fisher Scientific; Cat.-No. 00–4555-56) for 10 min. The remaining cells were detached using a cell scraper (Sarstedt). The cells were transferred to flow cytometry tubes, washed with PBS and stained in 100 µL Annexin binding buffer (0,14 M NaCl, 2,5 mM CaCl2, 0,01 M HEPES, pH 7,4) with 2 µL FITC-labeled Annexin V (Immunotools, Friesoythe, Germany; Cat.-No. 31490013) for 15 min at room temperature in the dark. The cells were then washed with 1 mL binding buffer and re-suspended in 100 µL binding buffer prior to measurement. For flow cytometry a FACSCanto II cytometer (BD, Franklin Lakes, USA) was used. Annexin-positive cells were identified by using sequential gating on single, viable cells based on forward and side scatter characteristics, followed by discrimination of Annexin-positive populations according to fluorescence intensity relative to unstained and single-stained controls. A positive control consisting of dead cells was included to verify Annexin staining and establish the gating strategy.

### Seahorse XF Cell Mito Stress Test

Primary murine microglia were seeded on 96-well Agilent Seahorse XF cell culture microplates (Agilent, Santa Clara, USA; Cat.-No. 102601-100) at a density of 60,000 cells/well. The cells were allowed to adhere for 24 h and then treated with the respective drugs for 24 h. The sensor cartridges were hydrated using the Seahorse XF Calibrant (Agilent; Cat.-No. 00840-000) at 37 °C overnight in a non-CO_2_-incubator. On the day of measurement, the microglia medium was exchanged to Seahorse Assay Medium (Agilent; Cat.-No. 103575-100) with 10 mM glucose (Agilent; Cat.-No.: 103577-100) 2 mM L-glutamine (Agilent, Cat.-No. 103579-100) and 2 mM sodium pyruvate (Agilent, Cat.-No. 103578-100). Cells were then incubated in a non-CO_2_- incubator for one hour.

Seahorse Bioscience XF96 Extracellular Flux Analyzer (Agilent Technologies) was used to perform the Cell Mito Stress Test as described by the manufacturer. Oligomycin (1 µM), FCCP (3 µM) and rotenone+antimycin A (0.5 µM/0.5 µM) (Agilent; Cat.-No. 103015-100) were subsequently injected into the wells according to the manufacturer’s protocol. For data analysis Wave Software (Agilent Technologies) was used.

### Reactive Oxygen Species Assay

The cellular production of reactive oxygen species was determined using the fluorogenic dye 2′,7′-dichlorodihydrofluorescein diacetate (H2DCFDA, Sigma Aldrich; Cat.-No. D6883) which diffuses across the plasma membrane and is intracellularly oxidized to a fluorescent product.

Microglia were seeded at a density of 150,000 cells per well on a 24-well plate. After 24 h of iron exposure, cells were harvested and stained for 30 min with 20 µM H2DCFDA and subjected to flow cytometry using the Alexa Fluor 488 channel on a FACSCanto II cytometer (BD). ROS production was quantified as mean fluorescence intensity per cell (MFI), which was obtained using FlowJo software (BD). The MFI values of each group were normalized to the mean MFI of the control group, which was set to 1. Accordingly, the presented data are expressed as relative fluorescence intensity.

### Quantification of Intracellular Iron by the Colorimetric Ferrozine Assay

The intracellular iron was quantified using a ferrozine-based colorimetric assay as previously described (Riemer et al. 2004) with slight modifications. Microglia were seeded with a density of 150,000 cells per well on 48-well plates. Cells were pre-treated for 24 h with DMSO or MMF, followed by a subsequent 24 h-exposure to Fe^2+^. After treatments, cells were washed twice with prewarmed PBS and then stored at −20 °C. The cells were lysed with 120 µL 50mM NaOH (Merck; Cat.-No. 1064621000). A part of the lysate was used for the quantification of protein with the bicinchonic acid assay (Thermo Fisher; Cat.-No. A55864). To the remaining lysates 100 µL of 10 mM HCl and 100 µL of a freshly prepared iron-releasing agent consisting of a 1:1 mixture of 1.4 M HCl and 4.5% KMnO_4_ (w/v) (Merck; Cat.-No. 1050820250) were added, and the plates were incubated for 2 h at 60 °C. After cooling to room temperature 37.5 µL of iron-detection reagent were added, containing 6.5 mM ferrozine (Sigma Aldrich, Cat.-No. 160601), 6.5 mM neocuproine (Sigma Aldrich; Cat.-No. N1501), 2.5 M ammonium acetate (Carl Roth; Cat.-No. 7869.1), and 1 M ascorbic acid (Sigma Aldrich, Cat.-No. 255564). The plates were then shaken for 30 min at room temperature. The 250 µL from each well were transferred to a 96-well plate and absorbance was measured at 562 nm on a CLARIOstar plate reader (BMG Labtech).

A standard curve was obtained by treating Fe^3+^ standard solutions in the same way.

Finally, the iron content per milligram protein was calculated.

### qPCR

RNA was extracted from microglia (150.000 cells/well) after 24 h of treatment with MMF 100 µM or DMSO as vehicle control using RNeasy Plus Mini Kit (Qiagen, Hilden, Germany; Cat. No. 74134) according to the manufacturer’s protocol. Concentration of RNA was determined using a NanoDrop ND-1000 (Thermo Scientific). cDNA-Synthesis was performed using the Promega kit with the manufacturer’s protocol. GoTag master mix was used to perform a qPCR on a QuantStudio 3 Real Time PCR system and analyzed using QuantStudio Design & Analysis Software v1.5.1 (Thermo Fisher). Specific forward and reverse primers for the respective genes were used as shown in Supplementary Table S1. The relative gene expression was analyzed using the Pfaffl method (Pfaffl 2001) using *Actb* and *Tbp* as housekeeping genes. A log2-transformation was performed prior to statistical analysis to achieve normally distributed data. For graphical representation, the log2 fold change (FC) was calculated as the difference between log2-transformed gene expression values.

### Data Analysis

All experiments were performed at least three times with three or more technical replicates in each experiment. No a priori sample size calculation was performed. No randomization or blinding was applied. Cells were assigned to treatment groups based on plate position to ensure equal distribution and minimize edge effects. Given the objective nature of the quantitative readouts (automated measurements), the risk of experimenter bias was considered minimal. Graphpad Prism 10.2.3 (GraphPad Software, Boston, USA) was used for the statistical analysis of the data. Data are presented as means from the individual experiments ± standard deviation (SD). The normality of the data was assessed by using the Shapiro-Wilk test. Homogeneity of variances was assessed using the Brown-Forsythe test (prior to performing a one-way ANOVA) and in all cases the assumption of homogeneity was met. Since all data were normally distributed, the significance of differences between experimental and control groups was analyzed using a paired *t*-test for comparison of two groups or one-way analysis of variance (ANOVA) for comparison of more than two groups, followed by Dunnett’s multiple comparisons test. *P*-values of *<* 0.05 were considered statistically significant. For experiments with fewer than five biological replicates, statistical testing was not conducted in accordance with the journal guidelines; results are therefore reported descriptively.

### Proteomics Analysis

#### Sample Preparation, Lysis and Digestion

To investigate the impact of MMF treatment on the proteome of microglial cells, primary murine microglia were seeded at a density of 60,000 cells per well in a 96-well plate and treated for 24 h with either 100 µM MMF or vehicle control. Subsequently, 30 µL of ammonium bicarbonate (AMBIC, AppliChem, Darmstadt, Germany; Cat.-No. A3583.0500) was added to each well, and the plates were stored at −80 °C until further use. Cell lysis was performed by adding n-Dodecyl β-D-maltoside (final concentration 0.03%, Thermo Fisher Scientific; Cat.-No. BN2005), followed by two consecutive freeze-thaw cycles at −80 °C and 40 °C, respectively. The lysates were digested into peptides using 0.05 µg trypsin (Serva, Heidelberg, Germany; Cat.-No 37286.04, Ratio Trypsin: Protein 1:40) per sample without prior reduction/alkylation steps. Digestion was carried out overnight at 37 °C in a ThermoMixer (Eppendorf) with gentle shaking (300 rpm) and was stopped by acidifying with trifluoroacetic acid (final concentration 0.1%; Carl Roth, Germany, Cat.-No. 1EHK.1).

#### Mass Spectrometry Analysis

For mass spectrometric analysis, approximately 200 ng of each sample was injected and concentrated on a precolumn (PepMap Neo C18 Trap Cartridge, 300 μm × 0.5 cm, particle size 5 μm, Thermo Fisher Scientific, Bremen, Germany; Cat.-No. 174500) using a Vanquish Neo UHPLC system (Thermo Fisher Scientific, Bremen, Germany). Peptides were subsequently separated on an analytical column (DNV PepMap™ Neo, 75 μm × 150 mm, C18, particle size 2 μm, pore size 100 Å, Thermo Fisher Scientifiic, Bremen, Germany; Cat.-No. DNV75150PN) at a flow rate of 400 nL/min. A solvent gradient of 1% to 21% solvent B (84% acetonitrile, Altmann Analytik, Germany; Cat.-No. MC1000292500 and 0.1% formic acid, Carl Roth; Cat.-No. 1EHN.2) was applied over 70 min, followed by an increase to 40% B by 95 min. The column was rinsed with 95% B for 5 min.

Peptides were ionized by electrospray ionization (ESI) prior to entering the Orbitrap Fusion Lumos mass spectrometer (Thermo Fisher Scientific, Bremen, Germany). The capillary temperature was set to 275 °C and the spray voltage to 1800 V. MS1 spectra were acquired in the range of 350–1000 m/z at a resolution of 120,000 (normalized AGC target: 100%, maximum injection time: 120 ms). For data-independent acquisition (DIA), 20 equally sized isolation windows were used to scan the 400–900 m/z range on the MS2 level. The Orbitrap resolution for MS2 was set to 30,000, and fragment ions were generated using higher-energy collisional dissociation (HCD) at a normalized collision energy of 32. The normalized AGC target was set to 1000%, with a maximum injection time of 54 ms. Replicates were measured in a randomized order, whereby control and treatment groups were always alternated. Between each sample the column was rinsed to prevent sample carry-over.

#### Data Analysis

The generated DIA data (raw format) were analyzed in the SpectronautPulsar program (version 19.5.241126.62635, Biognosys, Schlieren, Switzerland) employing the directDIA option. Analysis settings were used according to the manufacturer’s recommendations, with the exception that trypsin was specified as the digestion enzyme and oxidation on methionine was set as a variable modification. The mouse reference proteome from Uniprot (03_2025, 54,739 entries) was selected as the search database. Label free quantified (LFQ) values were further utilized for statistical analysis in Perseus (Tyanova et al. 2016). Stringent filtering criteria were applied, retaining only proteins that were quantified in at least 6 out of 9 replicates per group for further statistical evaluation. Remaining missing values were imputed from normal distribution utilizing a width of 0.3 and a down shift of 1.8. An unpaired Student’s *t*-test was performed, followed by multiple testing correction using the Benjamini-Hochberg procedure (FDR = 0.05) to identify significantly differentially expressed proteins. Proteins with an adjusted *p*-value < 0.05 were considered statistically significant. To determine the direction of regulation between two sample groups, the average log₂ LFQ values per protein for each group were subtracted from one another, resulting in a fold change (FC). Functional profiling of biological processes (BP), cellular components (CC), and molecular functions (MF), as well as pathway enrichment based on Reactome, KEGG, and WikiPathways, was performed using g: profiler (Kolberg et al. 2023). Only proteins that were more abundant in one comparison group and showed a fold change > 1.5 were assessed. To group mitochondrial and mitophagy proteins according to similarity of abundance profiles across different conditions unsupervised hierarchical clustering analysis was carried out. The distance metric was based on the Pearson correlation coefficient. The optimal cluster number was selected based on Silhouette values. The Z-scores for each protein were calculated and the results were visualized using a heat map. The data analysis and visualization was carried out using R version 4.4.1(R Core Team) using “ProtStatsWF” version 1.0.0 and “EnhancedVolcano” version 1.22.0 packages.

## Results

### MMF Increases Microglial Metabolic Activity in a Nrf2-Dependent Way

**Fig. 1:**
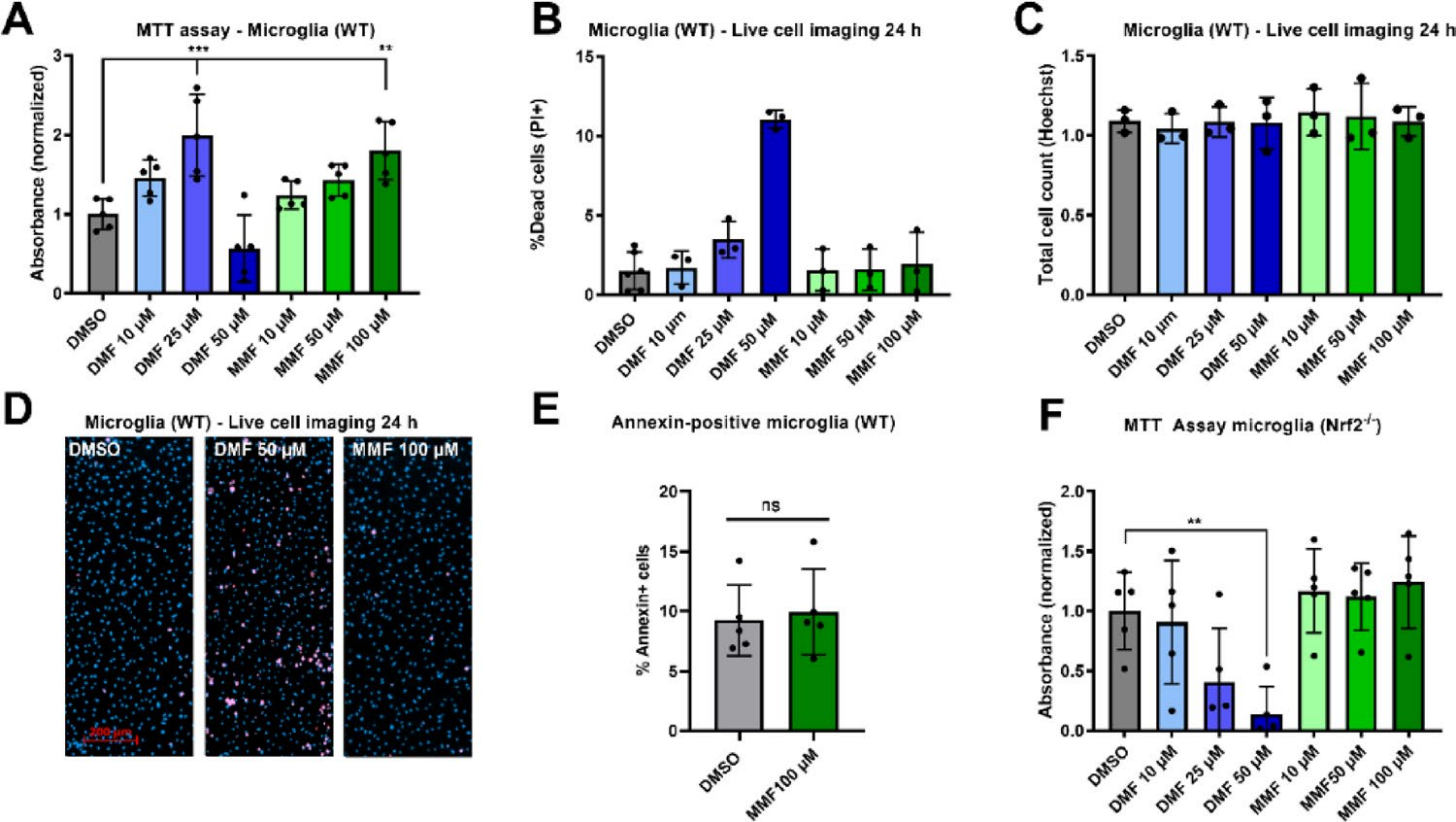
Effect of fumarates on viability and metabolic activity of microglia. Primary murine microglia cultures were treated for 24 h with various concentrations of MMF and DMF. MMF increases MTT reduction, whereas DMF exhibited a biphasic response **A** (*n* = 5 independent experiments). Live cell imaging of microglia using Hoechst 33,342 to determine the total cell number and propidium iodide (PI) to identify dead cells indicated apparent an increase in PI-positive cells following DMF treatment, whereas MMF showed no noticeable effect on cell death **B**. Total cell counts, including both live and dead cells, appeared unchanged across the treatment conditions, suggesting no obvious changes in cell proliferation (**C**) (*n* = 3 independent experiments). **D** shows representative images from one experiment with Hoechst 33,342 in blue and PI in red. A flowcytometric Annexin V assay demonstrated that there is no significant effect of MMF on early apoptotic stages that could explain the enhanced MTT reduction (**E**) (*n* = 5 independent experiments). The beneficial effect of MMF is attenuated in Nrf2^-/-^ microglia, while the DMF toxicity is more severe (**F**) (*n* = 5 independent experiments). All graphs represent the means ± SD, *p <.05, **p <.01, ***p <.001, ****p <.0001, as determined by one-way ANOVA (**A**, **F**) or paired *t*-test (**E**). No statistical analysis was performed for panels B and C in accordance with the journal guidelines, as fewer than five independent experiments were conducted (*n* < 5)

We first set out to investigate the effects of DMF and MMF treatment on cellular viability and metabolic activity as assessed by MTT assay. In primary murine microglia incubation for 24 h with MMF or DMF revealed significant differences in MTT conversion (*F*_(6, 28)_ = 10.97, *p* <.0001; Fig. 1A). MMF enhanced the MTT reduction of microglia in the MTT assay in a dose-dependent manner, with a significant increase at 100 µM compared to the DMSO control (*p* =.0029). In contrast, DMF increased MTT reduction only at 25 µM (*p* =.0003), whereas the higher concentration of 50 µM resulted in decreased MTT reduction (*p* =.1779). Using live cell imaging with PI staining to identify dead cells, DMF appeared to induce a dose-dependent toxic effect in microglia (Fig. 1B). In contrast, MMF-treated microglia showed no obvious differences in total cell number or cell death compared to control wells under the tested conditions (Fig. 1C). Representative images are shown in Fig. 1D. Only the conditions DMF 50 µM and MMF 100 µM are presented, as these concentrations produced the most pronounced effects in microglia, with DMF showing the strongest toxicity and MMF showing the strongest increase in MTT reduction – without toxicity. As Hoechst/PI staining did not account for the changes in MTT reduction following MMF treatment, an Annexin V assay was performed in MMF-treated microglia and DMSO controls, revealing no differences in early stages of cell death (t_(4)_ = 1.150, *p* =.3143; Fig. 1E). Together, these findings suggest that the increased MTT reduction observed in MMF-treated cells reflects enhanced metabolic activity rather than increased viability. Effects on primary murine astrocytes were less pronounced and are therefore shown in Supplementary Figure S4.

Next, we analyzed the effects of MMF and DMF on microglia from Nrf2^−/−^ mice (*F*_*(*6, 28)_ = 6.315, *p* =.0003; Fig. 1F). Here, the positive effect of MMF on MTT conversion was markedly attenuated, aligning with a higher vulnerability to the DMF-mediated toxic effect with significant toxicity at 50 µM (*p* =.0054).

### MMF Improves Mitochondrial Respiration

**Fig. 2:**
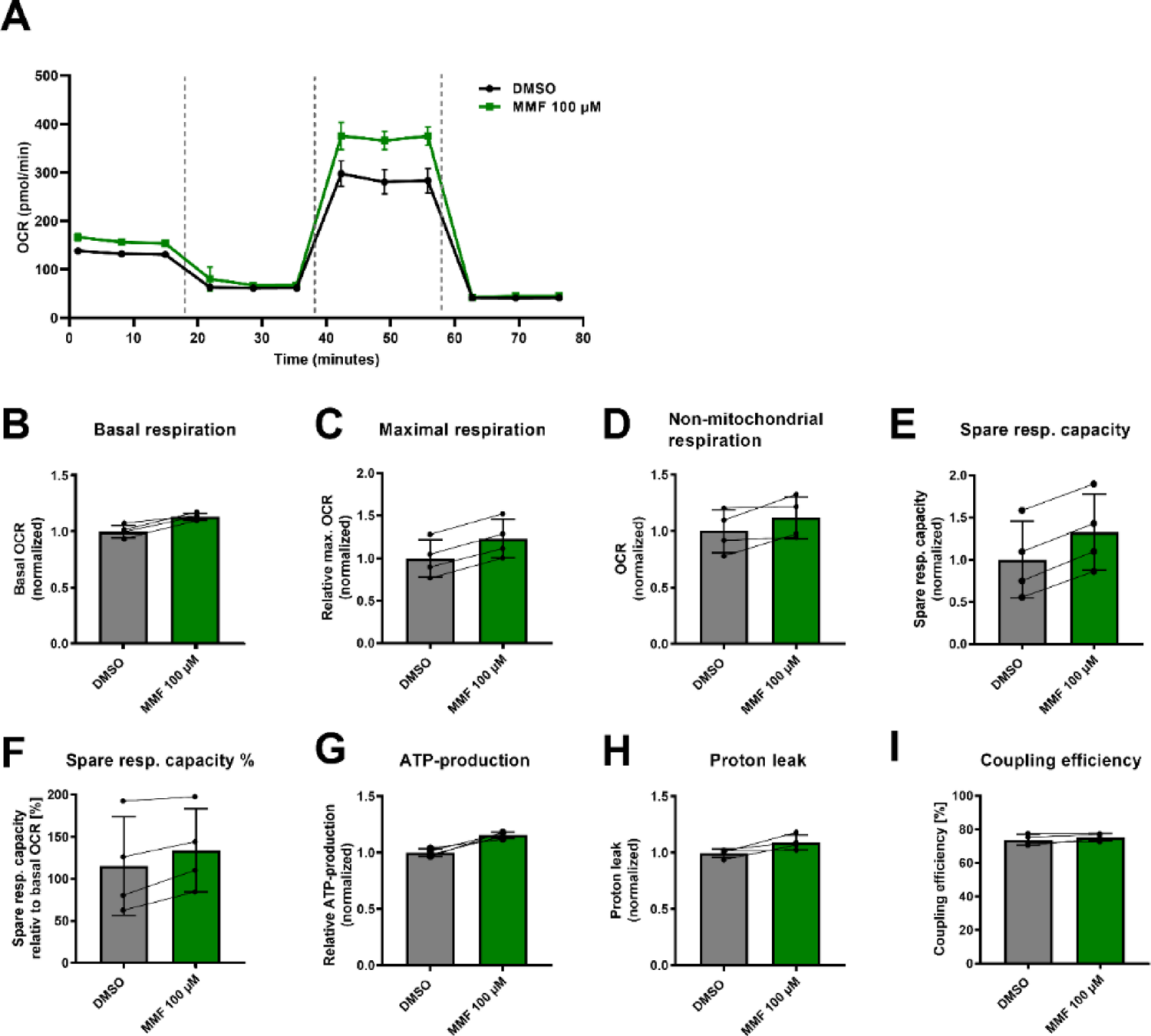
Mitochondrial response of microglia to monomethyl fumarate. Primary microglia were treated for 24 h with 100 µM MMF or vehicle. Afterwards Seahorse XF Cell Mito Stress Test was performed with subsequent injections of Oligomycin, FCCP and Rotenone/Antimycin A. **A** shows the OCR Data from one representative experiment carried out in quadruplicates. **B**–**I** show the respective parameters of mitochondrial bioenergetics from *n* = 4 independent experiments. All graphs represent the means ± SD. No statistical analysis was performed in accordance with the journal guidelines, as fewer than five independent experiments were conducted (*n* < 5)

To further investigate the effect of MMF on cellular bioenergetics, a Seahorse Mito Stress assay was performed in primary murine microglia. The oxygen consumption rate (OCR) was measured after the sequential injection of oligomycin, FCCP, and rotenone/antimycin (Fig. 2A). Although no statistical analysis was performed for these measurements in accordance with the journal guidelines, descriptive differences were observed between the groups. Compared to control cells, MMF-treated microglia showed higher basal respiration (Fig. 2B) and an increased maximal respiration following FCCP injection (Fig. 2C). In contrast, non-mitochondrial respiration after rotenone/antimycin A injection appeared largely unchanged (Fig. 2D).

**Fig. 3:**
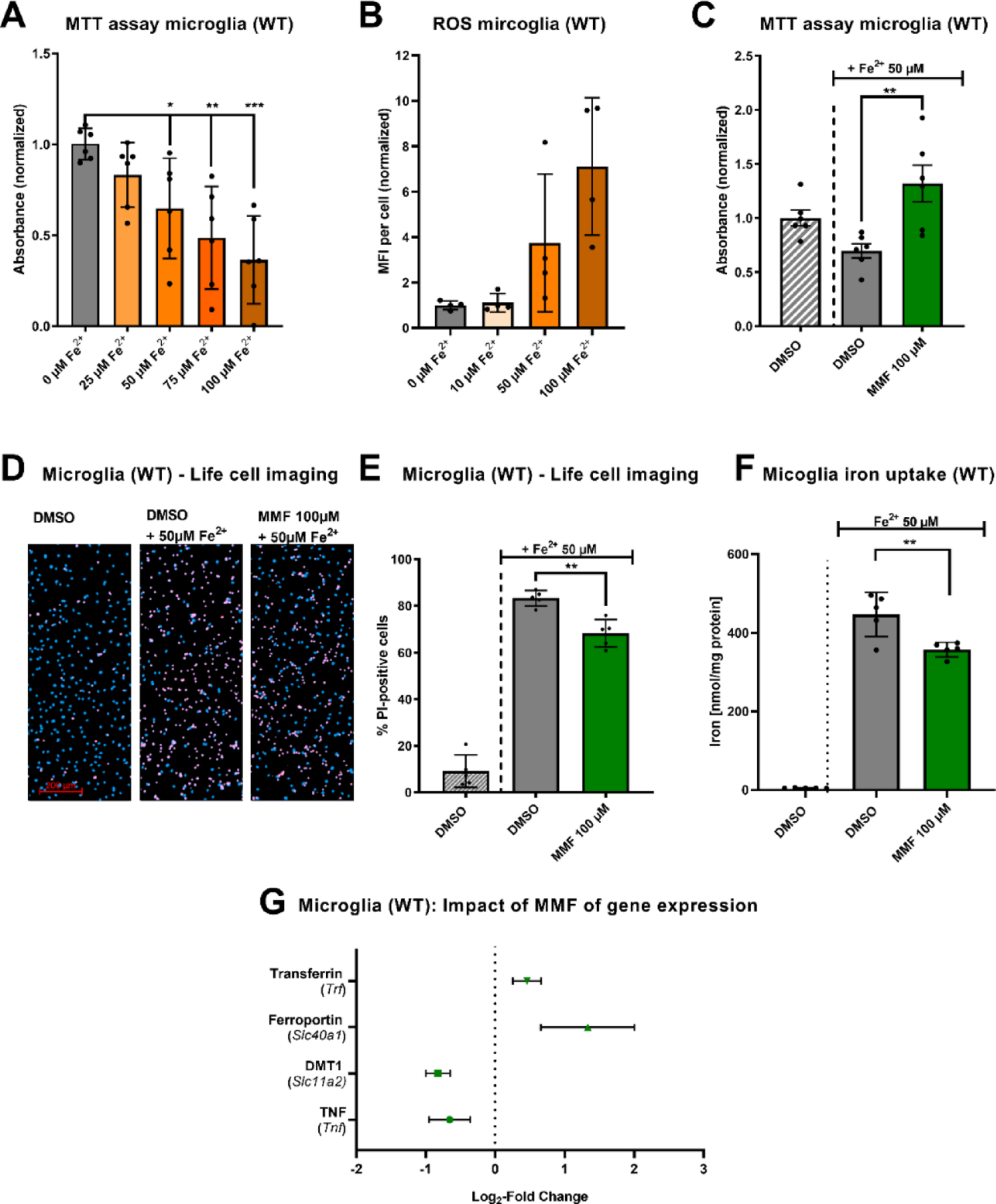
Monomethyl fumarate mitigates iron mediated effects of microglia. Treating primary microglia cell cultures for 24 h with increasing concentrations of Fe^2+^ leads to a dose-dependent decline in MTT reduction **A** (*n* = 6 experiments). Flow-cytometric measurement of ROS production using the H2DCFDA assay shows a dose-dependent increase in oxidative stress after 24 h of Fe^2+^ exposition (**B**) (*n* = 3 experiments). Pre-treating the cells with MMF for 24 h counteracts the detrimental effect of iron on microglial metabolic activity in the MTT assay (**C**) (*n* = 6 experiments). Hoechst 33,342/Propidium iodide staining shows that MMF reduces the toxic effect of Fe^2+^ on microglia (**E**) (*n* = 5 experiments), as shown by the representative images in **D**. Pre-treating microglia with MMF 24 h prior to Fe^2+^ exposition reduces iron uptake (**F**) (*n* = 5 experiments). Realtime-PCR data shows that MMF reduces the expression of the iron importer DMT1 (*Slc11a2*) and an upregulation of transferrin (*Trf*) and the iron exporter ferroportin (*Slc40a1*) while TNF (*Tnf*) is also downregulated (**G**) (*n* = 4 experiments). All graphs represent the means ± SD, *p <.05, **p <.01, ***p <.001, ****p <.0001, as determined by one-way ANOVA (**A**) or paired *t*-test (C, E, F). No statistical analysis was performed for panels **B** and **G** in accordance with the journal guidelines, as fewer than five independent experiments were conducted (*n* < 5)

From these values, further parameters were calculated: Spare respiratory capacity appeared elevated in MMF-treated cells (Fig. 2E), with the relative spare respiratory capacity, normalized to basal respiration, also showing a tendency toward an increase (Fig. 2F). ATP production was likewise higher in MMF-treated cells (Fig. 2G), whereas proton leak only showed a modest increase (Fig. 2H) and coupling efficiency was unchanged (Fig. 3I). Overall, these descriptive findings suggest an overall increase in mitochondrial respiration following MMF treatment. This observation could be attributed to either an increase in mitochondrial number or enhanced mitochondrial activity per organelle.

### Iron is Toxic to Microglia Through Reactive Oxygen Species

Since iron is critical for the activation of microglia and involved in the formation of paramagnetic rim lesions (PRLs), associated with progression, we set out to investigate effects of iron on microglia. When treating primary murine microglia with increasing concentrations of iron(II) sulfate there was a dose-dependent decline of MTT reduction as measured by using an MTT assay (*F*_(4, 25)_ = 7.817, *p* =.0003; Fig. 3A), accompanied by increasing cell death as shown with Hoechst 33,342/PI staining. This was consistent with elevated oxidative stress, as higher MMF concentrations were accompanied by visibly higher fluorescence signals in the flow cytometry-based H2DCFDA reactive oxygen species assay (Fig. 3B). The concentration of 50 µM Fe²⁺ was selected for subsequent experiments because it induced a moderate yet significant reduction in cell viability in the MTT assay, allowing the detection of both detrimental and potential protective effects. Pretreating microglia with MMF for 24 h prior to iron exposure counteracted the iron-induced decrease in MTT reduction and even increased MTT reduction beyond the level of cells not exposed to iron (*t*_(10)_ = 3.427, *p* =.0065; Fig. 3C). Hoechst 33,342/PI staining further revealed that MMF partially mitigated iron-induced cell death, although it did not completely prevent it (*t*_(8)_ = 4.991, *p* =.0011; Fig. 3D, E).

### MMF Reduces Microglial Iron Uptake and Alters Iron-metabolism

We further sought to investigate to what extend MMF modulates the cellular iron metabolism. To assess functional effects, we analyzed iron uptake in primary murine microglia using the colorimetric ferrozine assay. Treatment with 50 µM Fe^2+^ resulted in a 98-fold increase in cellular iron content. Pretreatment with 100 µM MMF reduced iron uptake by 20% (*t*_(8)_ = 3.386, *p* =.0096, Fig. 3F). To elucidate the underlying mechanisms, we analyzed the expression of key iron metabolism-related genes following treatment with 100 µM MMF compared to vehicle controls (Fig. 3G). Although no statistical analysis was performed for these measurements in accordance with the journal guidelines, descriptive differences in gene expression patterns were observed between MMF-treated and control samples. Expression of the iron exporter ferroportin (*Slc40a1*) and transferrin (*Trf*) appeared higher, whereas expression of the iron importer DMT1 (*Slc11a2*) and the pro-inflammatory cytokine TNF (*Tnf*) appeared lower in MMF-treated samples.

### Proteomic Analysis

**Fig. 4:**
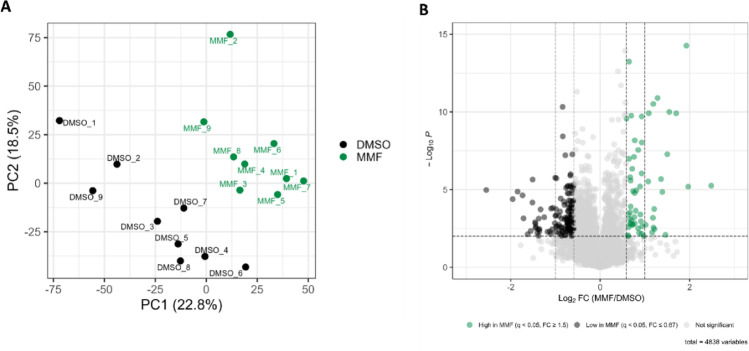
Proteomic analysis of MMF treated microglia. **A** Principial component analysis. DMSO and MMF treated cells form distinct clusters and can be distinguished in the PCA. **B** Volcano Plot displaying proteins being differential between DMSO and MMF treated cells. Proteins found to be of higher abundance in DMSO cells are highlighted in black and proteins being of higher abundance in MMF-treated cells in green (Student’s *t*-test adjusted *p*-value < 0.05). The horizontal dotted line indicates the *p*-value threshold and vertical dotted lines represent a log2 fold change cut-off of 0.58 and − 0.58 corresponding to a fold change of 1.5 or 0.67 and a log2 fold change cut off of 1 and − 1 corresponding to a fold change of 2 or 0.5 respectively

Mass spectrometric analysis of DMSO (Ctrl) and MMF-treated primary murine microglia (*n* = 9 per group) led to the quantification of 4,838 protein groups. To obtain an initial overview of whether MMF treatment alters the microglial proteome, principal component analysis (PCA) was performed (see Fig. 4A). The two conditions formed distinct clusters in the PCA, indicating a treatment-dependent effect of MMF on protein expression. Notably, sample MMF_2 clustered further apart from the other MMF-treated samples.

To identify proteins with altered abundance in response to MMF treatment, a relative comparison between the two groups was conducted (see Fig. 4B and Table S2). In total, 985 proteins were found to be differentially expressed (unpaired Student’s *t*-test, adjusted *p*-value < 0.05; Supplementary Data S1).

To gain insight into the biological processes associated with the differentially expressed proteins, functional profiling was performed. Proteins with higher abundance in DMSO-treated cells were predominantly associated with the respiratory chain, purine biosynthesis, and innate immune responses. In contrast, MMF-treated cells showed increased levels of proteins involved in oxidative or cellular stress responses, oxidoreductase activity, antioxidant functions, and pathways such as the Nrf2 signaling pathway.

These findings align with previous reports, as MMF is known to function as an immunoregulator with antioxidant properties, modulating the Nrf2 pathway (Linker et al. 2011).

To support our initial findings, we aimed to identify differentially regulated proteins associated with the aforementioned processes. In total, 581 proteins were more abundant in DMSO-treated cells, of which 30 showed a fold change (FC) greater than 2. Among these, 12 proteins were associated with immune responses and 9 with cell proliferation (Supplementary Data S1).

In MMF-treated cells, 404 proteins were found to be more abundant, with 19 proteins displaying an FC greater than 2. Notably, 5 of these proteins were associated with the Nrf2 pathway. In addition, 5 further Nrf2-related proteins were identified as more abundant in MMF-treated cells, albeit with lower fold changes.

Interestingly several Nrf2 pathway-associated proteins are also known to be relevant in the process of ferroptosis, as indicated by functional profiling, among them Glutamate-cysteine ligase and Heme oxygenase 1.

Next to proteins associated with the Nrf2 pathway, several proteins known to be involved in signaling pathways, such as the MAP2 kinase and the NF-kappa-B and the Wnt/beta-catenin signaling pathway were identified.

**Fig. 5:**
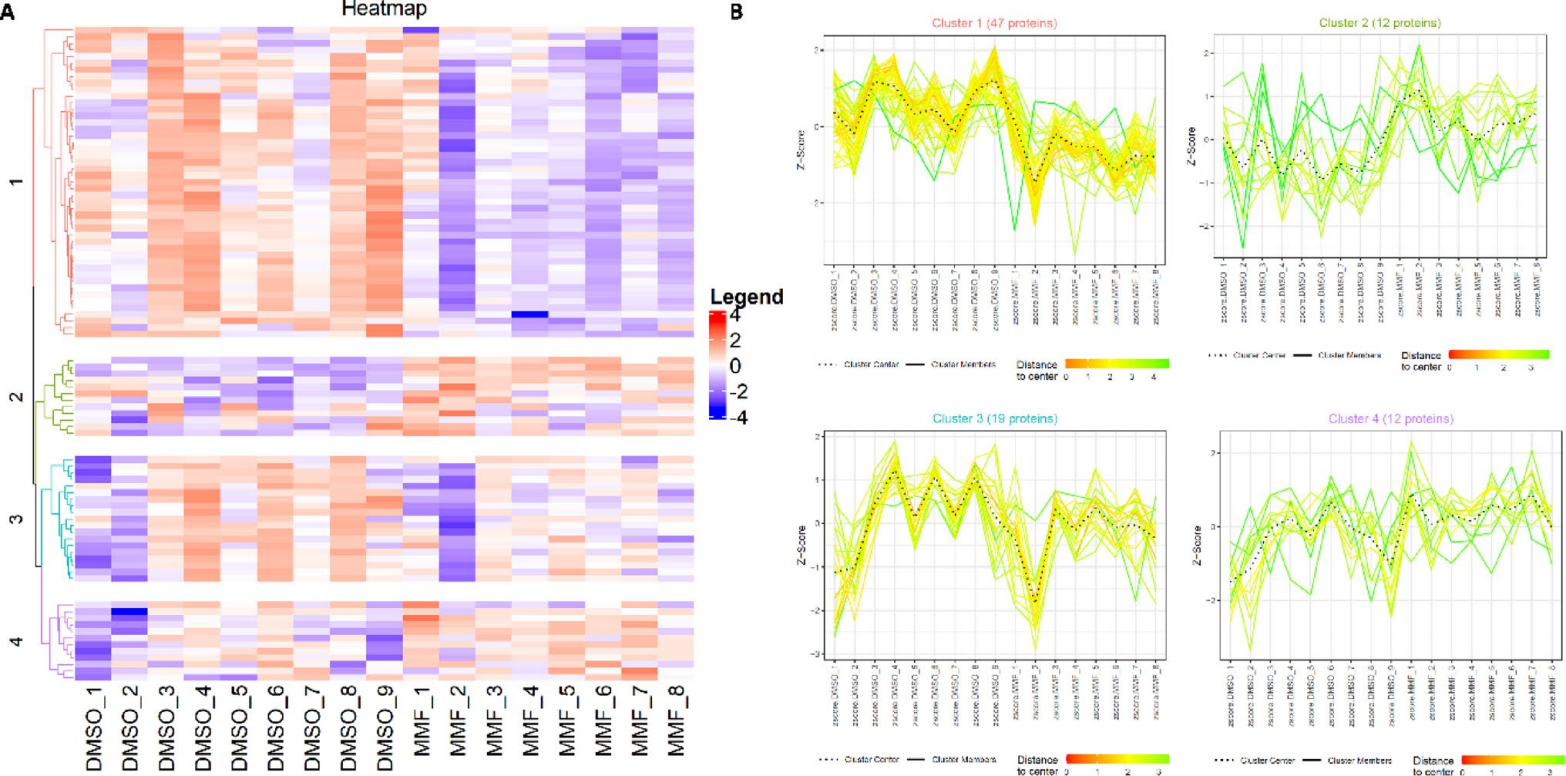
Heat map of respiratory chain proteins and mitophagy proteins. **A** Label free quantified protein values were z-scored and marked with a color-coded legend, whereby highest values are marked in red and lowest values in blue. The heat map was divided into four clusters. Line plots visualizing the abundances of proteins for each cluster and for each sample are shown in **B**

Building on previous findings indicating increased mitochondrial activity in MMF-treated cells, we subsequently aimed to characterize the expression profile of mitochondria-associated proteins. Interestingly, and contrary to expectations, DMSO-treated cells were found to display a higher abundance of 21 proteins associated with complex I, 2 proteins of complex II, 4 proteins of complex III, two proteins of complex IV and 4 proteins of complex V of the respiratory chain. In contrast, no proteins associated with any of the five complexes were found to be more abundant in MMF-treated cells (Supplementary Data S1). To understand protein dynamics of all respiratory chain associated proteins identified in the dataset, a heat map was created (Fig. 5). In total 38 proteins associated with complex I, 5 proteins of complex II, 8 proteins of complex III, 15 proteins of complex IV and 12 proteins associated with complex V could be identified in the whole data set. Additionally, 12 proteins known to be involved in mitophagy were utilized for clustering as well. Clustering of normalized label free quantified protein values (LFQ) was carried out, resulting in the identification of 4 clusters with different expression patterns, indicated as line plots (Fig. 5 and Supplementary Data S1). Cluster 1 contained 47 proteins with higher LFQ values in the DMSO group. In contrast, Cluster 2, comprising 12 proteins, showed the highest values in MMF-treated cells. Similar to Cluster 1, Cluster 3 (19 proteins) included proteins that were more abundant in DMSO-treated cells. In Cluster 4, LFQ values were also higher in MMF-treated cells, although the difference between the two groups was less pronounced than in Cluster 1. Notably, sample MMF_2 exhibited the lowest LFQ values for the majority of proteins. An in-depth analysis of all clusters revealed that proteins forming Clusters 1 and 3 were exclusively associated with the respiratory complexes. Notably, all mtDNA-encoded proteins were grouped into Cluster 3, except for cytochrome c oxidase subunit 3. In contrast, Clusters 2 and 4 were predominantly composed of proteins involved in mitophagy, such as reticulophagy regulator 1, BAG family molecular chaperone regulator 3, vacuolar protein sorting-associated protein 37B, sequestosome-1, parkinson disease protein 7 homolog, and transitional endoplasmic reticulum ATPase.

Regarding iron metabolism, we observed a higher abundance of the transferrin receptor and its regulator iron-responsive element binding protein 2, which is in line with previous findings indicating increased uptake of transferrin-bound iron (Pagani et al. 2020). Furthermore, we detected a higher abundance of the iron exporter natural resistance-associated macrophage protein 1 in MMF-treated cells. Consistent with our results from the iron toxicity assay, we found an upregulation of the ferroptosis suppressor protein. DMT1, ferroportin and ferritin were not detected by mass spectrometry.

## Discussion

In this study, we aimed to investigate the effects of DMF and MMF on microglia in the context of mitochondrial function and iron metabolism to further assess their neuroprotective mechanisms. Particular focus was placed on the effects of MMF, since DMF is a prodrug and does not naturally occur in the blood under physiological conditions. Nevertheless, we chose to investigate both compounds, to align our findings with the other publications that have examined both compounds or even just DMF and to highlight the necessity of distinguishing between DMF- and MMF-related effects in cell culture studies.

The findings of this work reveal that MMF increases MMT reduction of primary murine microglia in a dose-dependent manner, whereas its prodrug DMF exhibits a more biphasic effect: While low doses of DMF enhance the conversion of MTT - likely due to conversion to MMF - higher concentrations induced toxic effects, presumably as result of the glutathione depletion, as previously described (Albrecht et al. 2012).

Notably, neither total cell counts nor assays assessing cell death (Hoechst/PI and Annexin V staining) revealed differences between MMF-treated and control conditions, indicating that the increased MTT reduction is not attributable to changes in cell number or viability. Instead, these findings suggest that the enhanced MTT reduction primarily reflects increased cellular metabolic activity. Mechanistically, our experiments using Nrf2^−/−^ microglia suggest that the enhanced metabolic activity induced by MMF is at least partly dependent on the Nrf2 pathway, as these cells showed a markedly reduced response to MMF regarding MTT reduction. However, the presence of a non-significant trend even in the Nrf2^−/−^ cells suggests that additional pathways may also contribute to this effect.

The observed increase in MTT reduction is accompanied by enhanced mitochondrial respiration, as observed in the Seahorse XF Cell Mito Stress Test. The assay revealed an increase in basal and maximal respiration, spare respiratory capacity, proton leak and ATP-production without affecting the non-mitochondrial respiration. Given the fact, that inflammation impairs mitochondrial respiration (McCarthy et al. 2018) and mitochondrial dysfunction is considered a hallmark of progressive MS (Witte et al. 2014), these findings are of particular interest. The upregulation of mitochondrial respiration may help restore cellular energy production and thus contribute to the neuroprotective effects of fumaric acid esters.

To further elucidate the molecular mechanisms underlying the observed functional changes, we performed a comprehensive proteomic analysis comparing MMF-treated and DMSO-treated primary murine microglia. Surprisingly, despite the increased mitochondrial respiration observed in the Seahorse assay, the proteomic data did not support a corresponding upregulation of mitochondrial respiratory chain proteins in MMF-treated cells. In fact, proteins related to all five mitochondrial respiratory complexes (I–V) were found to be more abundant in DMSO-treated cells. These findings conflict with the reports by Hayashi et al. (2017), who demonstrated in both in vitro and in vivo studies that DMF-treatment increases mitochondrial biogenesis in various tissues, including the cerebellum, as evidenced by increased mtDNA content.

However, since we are unable to verify an increase in mitochondrial activity and further to trace mitochondrial protein synthesis and degradation without utilizing specialized mass spectrometry techniques, such as the incorporation of heavy labelled amino acids, the increase in mitochondrial respiration observed in the Seahorse assays may solely suggest an increased activity rather than an increase in mitochondria-associated proteins. Additionally, mitophagy-associated proteins were found to be of higher abundance in MMF treated cells, suggesting an increased turnover of mitochondrial proteins.

Our data thus indicate that the enhanced mitochondrial function observed in microglia following MMF treatment is unlikely to be solely driven by a classical increase in mitochondrial biogenesis or respiratory complex expression. Instead, the proteomic signature of MMF-treated cells pointed towards alternative mechanisms, such as an increased mitochondrial activity, potentially driven by enhanced mitophagy and thus increased protein turnover.

These observations suggest that MMF may enhance mitochondrial quality rather than quantity - possibly by promoting the removal of damaged mitochondria and maintaining a more functionally competent mitochondrial population. This interpretation is further supported by recent publications showing that the Nrf2 pathway activates mitophagy (Gumeni et al. 2021; Song et al. 2023; Chen et al. 2025).

Moreover, MMF-treated cells exhibited elevated levels of proteins associated with cellular stress responses, ferroptosis regulation, and major signaling pathways such as MAPK, NF-κB, and Wnt/β-catenin. These findings point to a broader adaptive cellular response that may collectively contribute to the preservation of mitochondrial function and enhanced cell viability under stress conditions. Together, these results suggest that MMF’s beneficial effects on mitochondrial respiration are mediated not through direct upregulation of mitochondrial structural components, but rather through qualitative remodeling of mitochondrial networks and activation of protective signaling cascades.

Another crucial aspect of this study was investigating the impact of MMF on iron metabolism, as chronically activated iron-loaded microglia have been shown to exert neurotoxic effects (Yauger et al. 2019; Dal-Bianco et al. 2021). Microglial cells treated with divalent iron exhibited a dose-dependent decrease in MTT reduction accompanied by an increase in cell death. In parallel, intracellular ROS production, as measured using the H2DCFDA assay, was elevated. Pretreatment of microglia with MMF prior to iron exposure increased MTT reduction and partially attenuated iron-mediated toxicity, as reflected by reduced cell death, thereby indicating a protective effect of MMF under conditions of iron overload.

In addition, MMF directly modulates microglial iron metabolism. Our qPCR-based gene expression showed differences in the expression patterns of iron metabolism-related genes between MMF-treated microglia and controls. Specifically, *Slc40a1* (ferroportin) appeared increased, whereas *Slc11a2* (DMT1) appeared reduced following MMF treatment. These observations are in line with our finding that iron uptake in microglial cells was reduced after MMF exposure in vitro. In addition, *Trf* expression appeared increased, which may indicate a shift toward transferrin-bound iron uptake, a pathway generally considered less toxic than non-transferrin-bound iron.

A comparable modulation of iron-related pathways in microglia has been reported for the anti-inflammatory cytokine IL-4 (McCarthy et al. 2018). The observations are in agreement with the works of Pagani et al. 2020; who have demonstrated an increase of transferrin uptake in microglia after treatment with DMF.

Supporting this, our proteomic analysis of MMF-treated microglia revealed a higher abundance of the transferrin receptor as well as its upstream regulator iron-responsive element binding protein 2, further indicating enhanced uptake of transferrin-bound iron. Additionally, we detected elevated levels of the iron exporter natural resistance-associated macrophage protein 1 in MMF-treated cells. In line with results from our iron toxicity assay, we also observed an upregulation of the ferroptosis suppressor protein, which may reflect a protective cellular response to altered iron homeostasis. Notably, neither ferroportin, DMT1, nor ferritin were detected by mass spectrometry, which may reflect technical limitations or low protein abundance under the experimental conditions applied.

While the results from this study are promising, there are several aspects that require further investigation. Especially the link between MMF treatment, mitochondrial function, and the Nrf2 pathway warrants more in-depth exploration to fully elucidate the interplay between these processes. Furthermore, an investigation of the role of the HCAR2 pathway in this context would complete the picture. As this study mainly focused on the effects of MMF on microglial cells, further investigation is needed to shed light on MMF’s effects on other CNS cell types, such as oligodendrocytes and astrocytes and the interplay between these cell types.

As for all in vitro data, the potential for translation of our findings to humans requires caution. For instance, the drug concentrations used in some of our experiments exceeded the physiologically detectable concentrations of MMF in the CSF. However, since in vitro conditions cannot fully replicate the complexities of a living organism, higher concentrations may be needed, to observe a clear biologic response. Especially given the fact that in vitro experiments only cover a period of a few days, whereas neuroprotective medications take a much longer time to exert effects in vivo. The results of this study support the growing body of evidence for the neuroprotective effects of MMF and highlight the role of microglial cells in this context.

By targeting iron-mediated toxicity and enhancing mitochondrial respiration, MMF demonstrates multifaceted beneficial effects. However, further research, including clinical trials in human MS patients, is needed to confirm MMF as a potential treatment for progressive MS. Nonetheless, the findings presented here contribute valuable insights into the development of novel therapeutic strategies for this challenging and debilitating disease.

## Supplementary Information

Below is the link to the electronic supplementary material.

Supplementary Data S1 Proteomics

Supplementary Methods and Figures

Supplementary Data S2 Statistics

## Data Availability

All the data are available from the corresponding author SF upon reasonable request.
