# Supplementary material for: Monomethyl Fumarate Modulates Iron Metabolism and Mitochondrial Function in Microglia with Implications for Multiple Sclerosis Progression
Source: Cell Mol Neurobiol. 2026 Jul 20;46:118. doi: 10.1007/s10571-026-01775-x (PMC13385331; doi:10.1007/s10571-026-01775-x)
Supplement: Supplementary file 2 — Supplementary Methods and Figures [file 10571_2026_1775_MOESM2_ESM.docx]

# **Monomethyl Fumarate Modulates Iron Metabolism and Mitochondrial Function in Microglia with Implications for Multiple Sclerosis Progression**

Justus Dann^1^, Katharina Klöster^1^, Ulas Ceylan^1^, Neele Heitmann^1^, Britta Eggers^2,3^, Svitlana Rozanova^2,3,4^, Martin Eisenacher^2,3,4^, Katrin Marcus^2,3^, Konstanze F. Winklhofer^5^, Ralf Gold^1^, Simon Faissner^1^

^1^ Department of Neurology, Ruhr-University Bochum, St. Josef-Hospital, Bochum, Germany

^2^ Medizinisches Proteom-Center, Medical Faculty, Ruhr-University Bochum, Bochum, Germany

^3^ Medical Proteome Analysis, Center for Protein Diagnostics (PRODI), Ruhr-University Bochum, Bochum, Germany

^4^ Core Unit for Bioinformatics (CUBiMed.RUB), Medical Faculty, Ruhr-University Bochum, Bochum, Germany

^5^ Department Molecular Cell Biology, Institute of Biochemistry and Pathobiochemistry, Ruhr-University Bochum, Bochum, Germany

**Corresponding author:** Prof. Dr. Simon Faissner, MD, [simon.faissner@rub.de](mailto:simon.faissner@rub.de)

**Supplementary file S1**

**Assessment of the purity of primary microglial cultures**

**
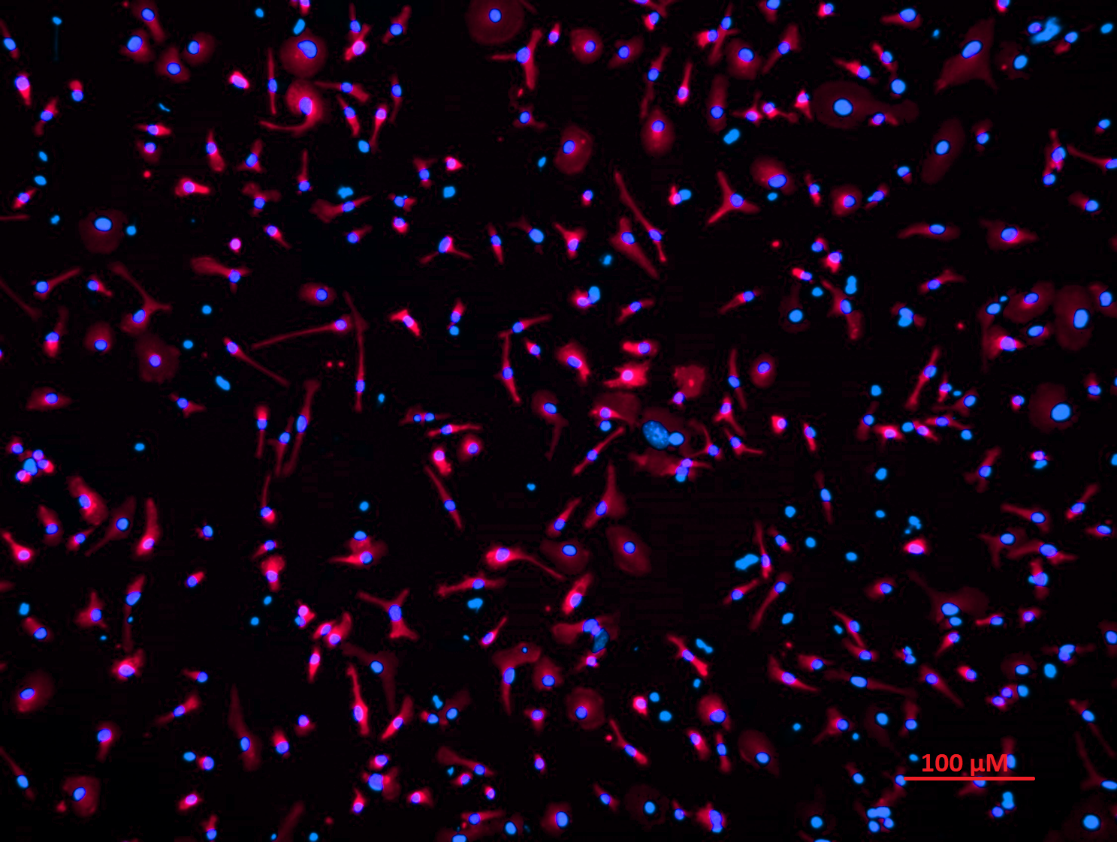
**

**Supplementary Figure S1:** Representative image of primary microglia stained for Iba1 (red) and DAPI. In total, four microscopic fields at 20× magnification were analyzed, with at least 500 cells counted overall. Cell purity was >95%.


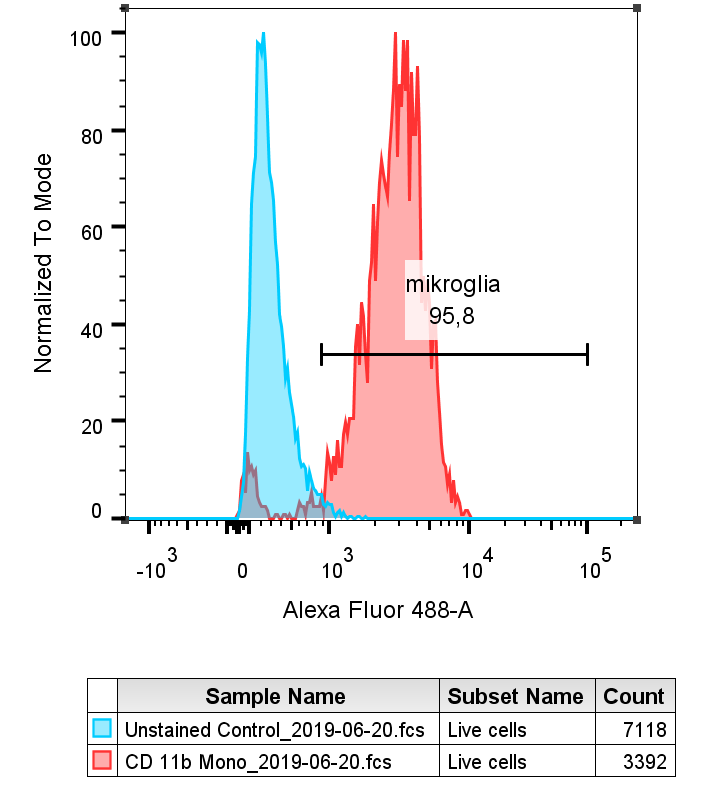
**qPCR primers**

**Supplementary Figure S2:** Representative flow cytometry analysis using CD11b staining. More than 95% of cells were CD11b-positive.

| **Gene** | **Sequence** |
| --- | --- |
| *Tnf* (TNF) | F: 5´- ATG GCC TCC CTC TCA TCA GT -3´  R: 5´- TGG TTT GCT ACG ACG TGG G -3´ |
| *Slc11a2* (DMT1) | F: 5´- AAA GAT GCC AGA CGA TGG CG -3´  R: 5´- ATC CGT GGG ACC TTG GGA TA -3´ |
| *Trf* (transferrin) | F: 5´- AGC CGA TGC TAT GAC CTT GG -3´  R: 5´- ACT GCC CGA GAA GAA ACT GG -3´ |
| *Slc40a1* (ferroportin) | F: 5´- GGC ACT TTG CAG TGT CTG TG -3´  R: 5´- GTG ACG TCT GGG CCA CTT TA -3´ |
| *Tbp* (TATA-binding protein) | F: 5´- AGC TCT GGA ATT GTA CCG CA -3´  R: 5´- TGA CTG CAG CAA ATC GCT TG -3´ |
| *Actb* (beta-actin) | F: 5´- GAC CTC TAT GCC AAC ACA GT -3´  R: 5´- AGT ACT TGC GCT CAG GAG GA-3´ |

**Supplementary Table S1:** Primer sequencing used for qPCR, including the housekeeping genes Tbp and Actb

**Fumarate effects on primary murine astrocytes**

**Preparation of primary murine astrocyte cultures**

Mixed glial cell cultures were prepared as described for microglia in the methods section. To obtain highly pure astrocytes, we used a shaking protocol previously described for rat astrocytes (Bettegazzi et al. 2011). The mixed glial cell culture flasks were shaken for 24 h at 200 rpm on day 2 and day 6 to remove any non-adhering cells. After 14 days the astrocytes were dissociated with Trypsin-EDTA 0.25% (GIBCO, Thermo Fisher Scientific; Cat.-No. 25200056) and then plated into poly-D-lysine-coated plates. In addition to the typical flat morphology observed by light microscopy, GFAP staining (Dako/Agilent, Cat.-No. Z0334) was performed to confirm the astrocytic identity of the cultured cells. Microglial contamination was assessed by Iba1 staining and revealed less than 1% Iba1-positive cells, corresponding to an astrocyte purity of >99% (see Fig. S3). Astrocytes were allowed to adhere for one week before experiments to ensure a mature phenotype. Astrocytes were seeded at a density of 20.000 cells per well and were allowed to adhere for one week before experiments to ensure a mature phenotype.


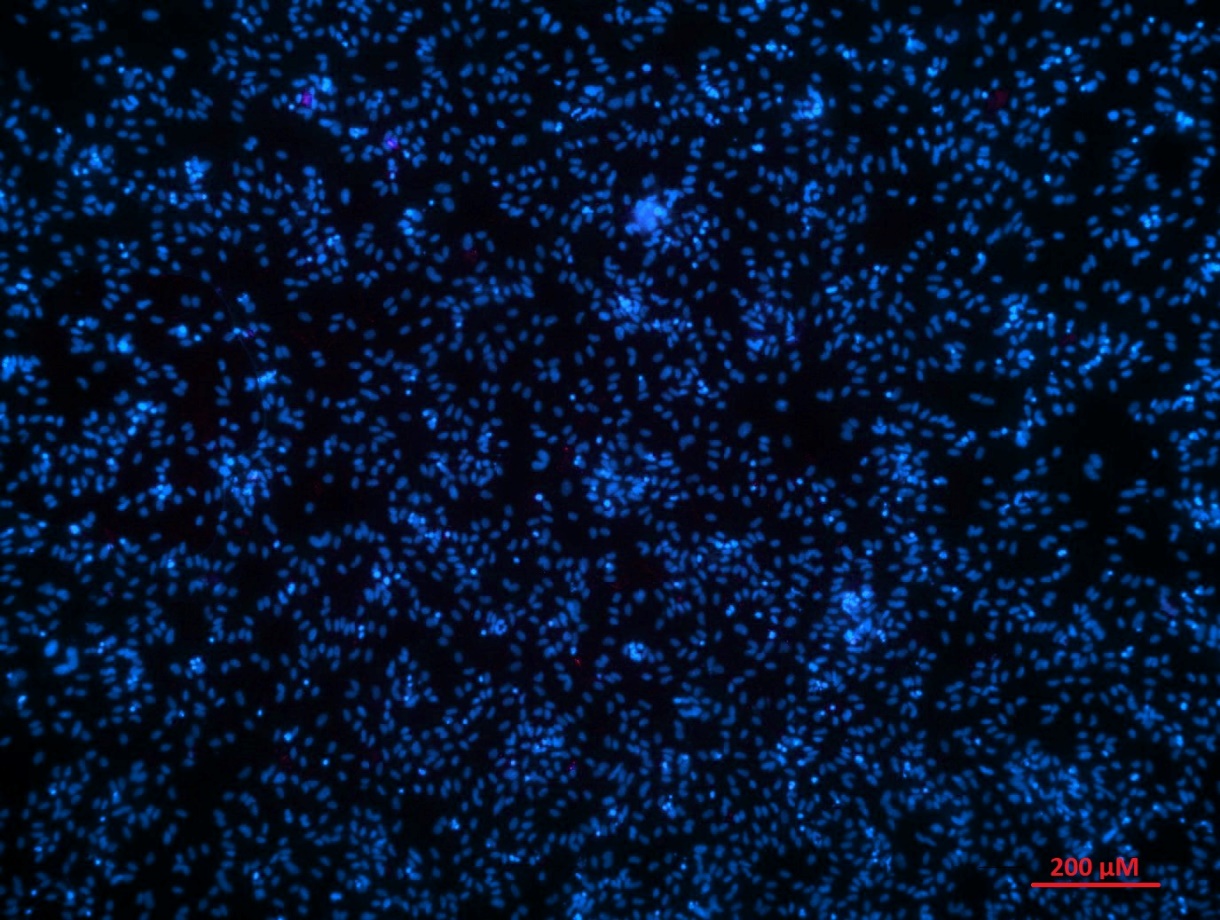

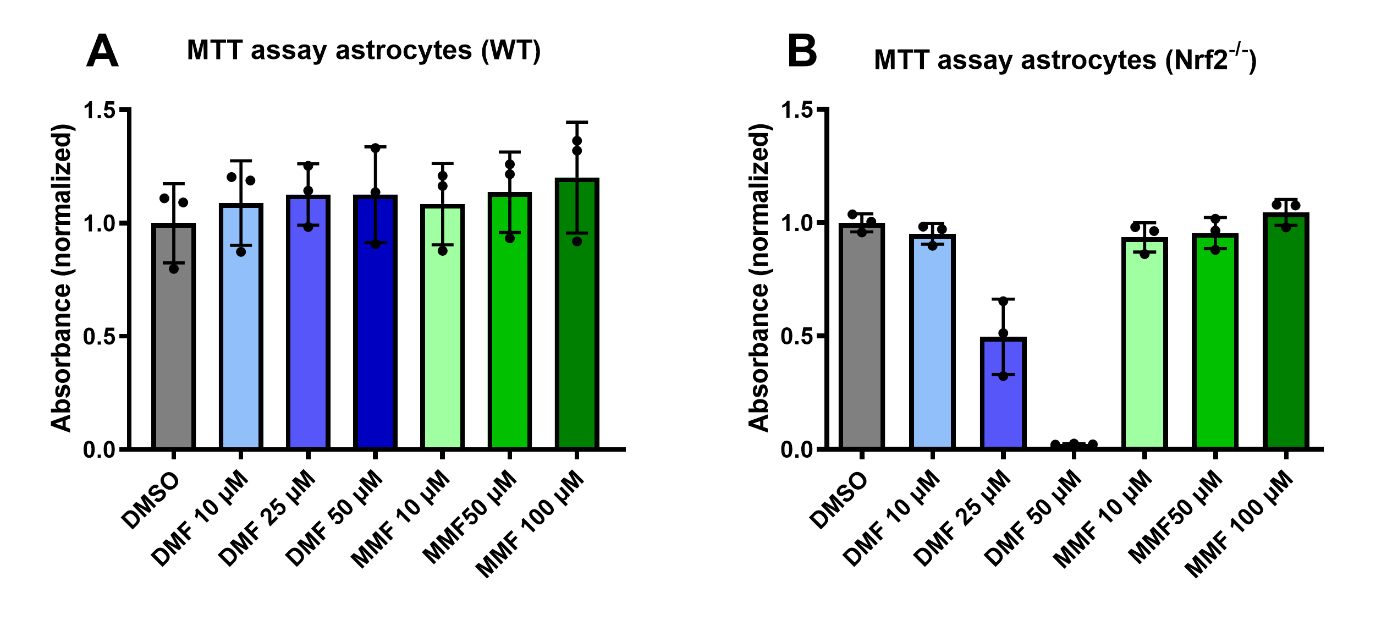


**Supplementary Figure S3**: Iba1/DAPI staining of primary astrocyte culture showing hardly any microglial contamination (red).

**Supplementary Figure S4**: In primary murine wildtype astrocyte cultures, MTT reduction appeared largely unchanged following treatment with fumaric acid esters (A) (n = 3 independent experiments). In contrast, Nrf2^-/-^ astrocytes showed an apparent increased susceptibility to DMF treatment at concentrations of 25 µM and 50 µM (B) (n = 3 independent experiments). All graphs represent means ± SD. No statistical analysis was performed due to the low number of independent experiments (n < 5), in accordance with the journal guidelines.

**References**
Bettegazzi B, Mihailovich M, Di Cesare A, Consonni A, Macco R, Pelizzoni I, Codazzi F, Grohovaz F, Zacchetti D (2011) β-Secretase activity in rat astrocytes: translational block of BACE1 and modulation of BACE2 expression. Eur J Neurosci 33:236–243. https://doi.org/10.1111/j.1460-9568.2010.07482.x
